# Jasmonate-Mediated Mitigation of Salinity Stress During Germination and Early Vegetative Development in Hemp

**DOI:** 10.3390/plants14182864

**Published:** 2025-09-15

**Authors:** Franciszek Kasprowiak, Emilia Wilmowicz, Agata Kućko

**Affiliations:** 1 Department of Plant Physiology, Institute of Biology, Warsaw University of Life Sciences-SGGW, Nowoursynowska 159, 02-776 Warsaw, Poland; s205127@sggw.edu.pl; 2 Department of Plant Physiology and Biotechnology, Faculty of Biological and Veterinary Sciences, Nicolaus Copernicus University, 1 Lwowska Street, 87-100 Toruń, Poland; emwil@umk.pl

**Keywords:** jasmonates, germination, hemp, stress tolerance, salinity

## Abstract

Climate change and soil salinization threaten crop productivity, particularly affecting salt-sensitive species like hemp (*Cannabis sativa* L.), which is gaining importance in sustainable agriculture and bioeconomy. Jasmonates (JAs) offer promising potential for enhancing plant abiotic stress tolerance. Given hemp’s inherently low salt tolerance and limited data on JAs-mediated responses, we investigated salinity tolerance JAs modulation using methyl jasmonate (MeJA; 0.001–0.01 mM) and the JAs-biosynthesis inhibitor mefenamic acid (MEF; 0.01–0.1 mM) applied via seed priming or foliar treatment in factorial experiments with NaCl concentrations of 0.05–0.3 M. We demonstrate that MeJA and MEF differentially modulate responses of Henola hemp variety to salt stress during germination and seedling development. At 0.1 M NaCl, 0.01 mM MeJA enhanced germination rate by 25% compared to the salt-only control, indicating a protective effect on initial development, whereas 0.1 mM MEF exacerbated salt toxicity by increasing seed damage and reducing respiration by 57%, subsequently suppressing seedling growth. In 25-day-old seedlings exposed to 0.3 M NaCl, 0.001 mM MeJA treatment increased root length by 30 mm, fresh biomass by 50%, chlorophyll content by 20%, and photosynthetic efficiency by 23%, while reducing water deficit by 60% and leaf injury by 40%. MEF co-treatment partially reversed these protective effects, reducing MeJA-mediated improvements, confirming that maintaining JAs homeostasis is critical for salt-stress adaptation. These findings establish MeJA as a promising tool for enhancing hemp cultivation under saline conditions and provide a framework for integrating JAs treatments into sustainable hemp cultivation protocols.

## 1. Introduction

Excessive soil salinization has emerged as a critical constraint to global agricultural productivity, currently affecting over 10% of arable land worldwide, reducing crop yields, threatening ecosystem integrity, and rising under climate-change pressures. According to FAO projections, within the next 25 years, up to 30% of currently cultivated land will become unproductive due to salinity, with the proportion potentially approaching 50% by the mid-21st century [1]. The developmental stage of plants critically determines their sensitivity to salt stress: early ontogenetic phases, seed germination, and seedling establishment are particularly vulnerable to elevated salinity [2,3]. High soil concentrations of Na^+^ and Cl^−^ disrupt cellular water potential and ion homeostasis, impairing water and nutrient uptake, and trigger overproduction of reactive oxygen species (ROS), resulting in combined oxidative and osmotic stresses [4]. Such disturbances reduce seed germination vigor, delay emergence, damage the embryo, and inhibit vegetative growth, ultimately curtailing organic matter accumulation and crop yield [5]. Concurrently, salt-induced perturbations in water relations elevate the content of stress-related phytohormones, such as jasmonates (JAs), ethylene (ET), or abscisic acid (ABA), thereby amplifying stress responses [6]. It should be noted, however, that the extent of damage depends on the adaptive capacity of individual plant species.

Among crop species, hemp (*Cannabis sativa* L.) combines broad stress tolerance with minimal agronomic inputs, making it well-suited for marginal environments. It yields high-quality bast fibers for textile [7], paper [8] construction, or automotive industries [9]; protein- and polyunsaturated fatty acid-rich seeds for food/feed [10]; and hemp seed oil for culinary, technical uses, and cosmetic applications [11]. Rapid growth and substantial biomass accumulation underpin its use in phytoremediation of contaminated soils, while efficient water and nutrient use enhance its sustainability [12]. These attributes—paired with emerging pharmaceutical uses of cannabidiol (CBD)-rich cultivars [13]—position hemp as a climate-smart multipurpose crop for resilient agricultural systems [14].

Given the rapid growth of the global population and the finite availability of arable land, developing effective strategies to counteract soil degradation—particularly salinization—is critical for sustaining agricultural productivity. This challenge is especially acute in economically important crops that must be cultivated on salt-affected soils [15]. The urgent need for solutions has prompted research into innovative agronomic approaches, notably seed priming and exogenous application of phytohormones, or their biosynthesis/signaling inhibitors, to mitigate the adverse effects of salinity and preserve crop yield and quality. JAs have emerged as a promising means to enhance salt tolerance [16].

In general, JAs exert broad-spectrum effects at every stage of plant development, orchestrating morphological, physiological, cellular, and molecular responses—from seed germination and hypocotyl elongation, stamen differentiation, reproductive development, apical hook growth, to stomatal regulation and senescence [17]. Jasmonic acid (JA) was initially characterized by its ability to suppress Arabidopsis root elongation [18]. However, the effects of JAs are highly context-dependent, with their beneficial or detrimental impacts determined by concentration, timing, plant developmental stage, and environmental conditions. At optimal concentrations, JAs coordinate defense responses under diverse abiotic stresses [19,20] and play a crucial role in wounding responses by inducing defensive proteinase inhibitor proteins, which block digestion and protect damaged tissues, e.g., in tomato [21]. However, high JAs doses can inhibit growth, reduce photosynthesis, and accelerate senescence [22,23,24], reflecting inherent trade-offs between growth and defense mechanisms. These phytohormones are central regulators of salt stress adaptation, accumulating in response to NaCl in species such as *Iris hexagona* [25] or common centaury [26]. In the halophyte *Solanum chilense*, JAs levels were more than double those observed in the glycophyte *S. lycopersicum*, and salinity stress further boosted their concentrations by over 100% [27]. When applied at appropriate concentrations, JAs have been shown to mitigate salt injury in *A. thaliana* [28], *Limonium bicolor* [29], and numerous crops, including rice [30], wheat [31], tomato [32], and sorghum [33]. Under saline conditions, induction of JAs biosynthetic genes (*LOX3*, *AOC*) and the accumulation of JA and its derivatives activate downstream regulators (MYC2, JAZ), thereby enhancing stress tolerance [34,35,36,37,38].

Among JAs derivatives, MeJA is distinguished by its high volatility, which facilitates long-distance transport, and its ability to promote photoassimilate translocation through vascular tissues—traits that enhance its effectiveness in mitigating salt-induced damage [39,40]. Accordingly, MeJA more robustly activates the JAs signaling cascade and induces JAs-responsive gene expression than equivalent concentrations of free JA [41]. In strawberry and *Arabidopsis thaliana*, MeJA treatment elevates anthocyanin levels, boosts antioxidant capacity, and upregulates key antioxidant enzymes [42,43], thereby reinforcing cellular homeostasis under salt stress. MeJA also stimulates osmoprotectant biosynthesis (e.g., proline), further stabilizing osmotic balance [44].

The response of the widely cultivated hemp variety Henola in Poland to JAs under salinity stress during early growth stages, which are critical as they determine subsequent development and ultimate yield, remains uncharacterized. Elucidating this response will inform agronomic strategies to enhance stress tolerance and productivity in salt-affected soils. Accordingly, this study tests the hypothesis that JAs can counteract salt-induced damage in young hemp by modulating physiological stress responses. By filling this knowledge gap, our findings aim to pave the way for reliable hemp cultivation on saline soils using MeJA treatments and thereby expand production capacity to meet growing market demand.

## 2. Results

### 2.1. Effectiveness of Salt Stress on Hemp Development

To assess the sensitivity of hemp to salinity during seed germination and vegetative growth, we performed germination assays and conducted treatments on 25-day-old plants. As shown in Supplementary Figure S1, increasing NaCl concentrations (0.1 M and 0.2 M) severely inhibited germination and reduced root and shoot growth. Notably, growth parameters were not significantly affected when seeds were incubated in 0.05 M NaCl solution. In contrast, at 0.3 M NaCl, germination was completely suppressed. These responses guided the selection of two NaCl concentrations (0.1 M and 0.2 M), which impaired but did not entirely inhibit seed germination and seedling development (Supplementary Figure S1). Accordingly, these solutions were subsequently used in further experiments to investigate the role of JAs in counteracting the effects of salinity during seed germination.

The detrimental effect of salt treatment on 25-day-old hemp plants became evident at NaCl concentrations of 0.2 M and 0.3 M, manifested by visible chlorotic discoloration and necrosis along leaf margins, as well as premature senescence of cotyledons (Supplementary Figure S2). Significant inhibition of shoot growth was observed, with reductions of ~4 cm and ~6 cm compared to the control at 0.2 M and 0.3 M, respectively. Importantly, all NaCl treatments caused significant inhibition of root growth, with the greatest physiological impact—a ~44% decrease in root length—recorded at 0.3 M NaCl (Supplementary Figure S2). Consequently, that concentration was selected for subsequent studies to investigate the role of JAs in modulating hemp growth under salinity stress during the vegetative stage.

### 2.2. Modulation of Hemp Seed Germination and Early Growth by MeJA and MEF Under Salt Stress

**Figure 1:**
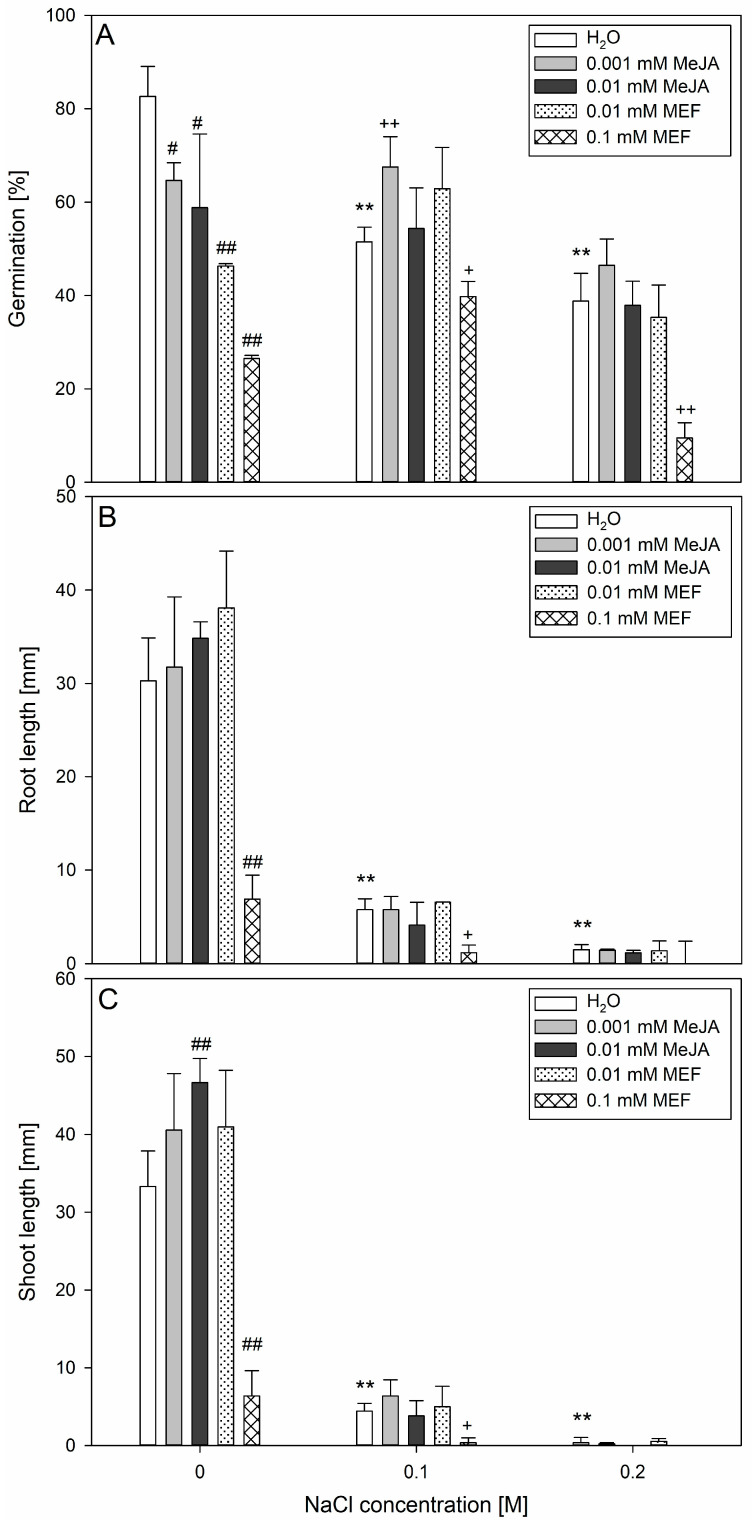
Effects of methyl jasmonate (MeJA) and mefenamic acid (MEF) on hemp seed germination and early seedling growth under salinity. Germination percentage (**A**), root (**B**), and shoot (**C**) length were measured on day 7 of the germination assay. Treatments included different concentrations of NaCl (0.1 M and 0.2 M), MeJA (0.001 mM and 0.01 mM), MEF (0.01 mM and 0.1 mM), and their combinations. Values represent means ± SD. Statistical significance: NaCl vs. H_2_O ** *p* ≤ 0.01; MeJA/MEF vs. H_2_O ^##^ *p* ≤ 0.01, ^#^ *p* ≤ 0.05; MeJA/MEF + NaCl vs. NaCl ^++^ *p* ≤ 0.01, ^+^ *p* ≤ 0.05.

Both MeJA and MEF negatively affected seed germination in hemp, with MEF exerting a stronger inhibitory effect (Figure 1A). The suppression of germination by the JAs biosynthesis inhibitor was concentration-dependent, resulting in up to a ~60% decrease at the highest applied dose (0.1 mM). MeJA significantly inhibited germination at both tested concentrations, leading to similar physiological responses. When seeds were treated with MeJA or MEF in combination with salt stress, the effects varied. Application of 0.001 mM MeJA alongside 0.1 M NaCl improved germination by approximately 25% compared to NaCl alone, though this positive influence was not observed at higher salinity levels. In contrast, 0.1 mM MEF amplified the detrimental effects of 0.1 M NaCl and 0.2 M NaCl, causing a further reduction in germination compared to salt stress alone. Importantly, this effect deepened with increasing solution concentration.

Assessment on day 7 revealed that NaCl treatments limited both shoot and root elongation in seedlings (Figure 1B,C). However, treatment with 0.01 mM MeJA significantly stimulated shoot elongation by ~25%, relative to the untreated control, without affecting root length, while 0.1 mM MEF severely inhibited both shoot and root growth. Importantly, simultaneous application of salinity and MeJA did not result in significant differences in seedling shoot or root length compared to salt-only treatments, regardless of the concentrations applied. Conversely, MEF at higher concentrations reduced the growth of both root and shoot under lower salinity conditions. These results highlight the differential effects of MeJA and MEF in modulating germination and early seedling growth of hemp under salt stress, with MEF generally exacerbating inhibitory effects and MeJA providing partial mitigation only at moderate salinity levels.

### 2.3. Jasmonate Modulation Alters Seed Damage and Respiration Caused by Salt Stress

**Figure 2:**
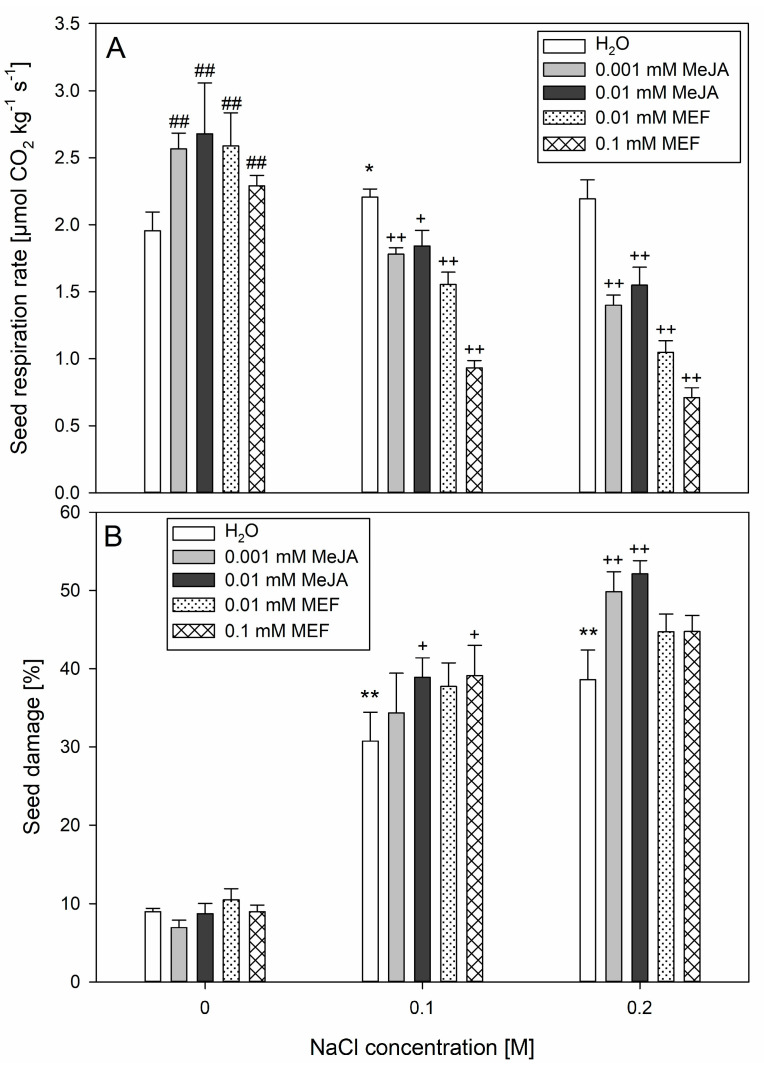
Effects of methyl jasmonate (MeJA) and mefenamic acid (MEF) on hemp seed respiration rate (**A**) and seed damage percentage (**B**) under salt stress. Seeds for the measurements were collected 24 h after incubation in NaCl (0.1 M and 0.2 M), MeJA (0.001 mM and 0.01 mM), or MEF (0.01 mM and 0.1 mM), and their mixtures. Values represent means ± SD. Statistical significance: NaCl vs. H_2_O ** *p* ≤ 0.01, * *p* ≤ 0.05; MeJA/MEF vs. H_2_O ^##^ *p* ≤ 0.01; MeJA/MEF + NaCl vs. NaCl ^++^ *p* ≤ 0.01, ^+^ *p* ≤ 0.05.

Respiration intensity analyses showed that treatments with 0.001 mM, 0.01 mM MeJA, as well as 0.01 mM MEF and 0.1 mM MEF, stimulated seed respiration by approximately 24%, 28%, 25%, and 15%, respectively, compared to untreated controls (Figure 2A). However, this stimulatory effect was counteracted by salt application. Co-application of 0.001 mM or 0.01 mM MeJA with 0.1 M NaCl led to a ~15% decrease in respiration intensity relative to seeds treated with 0.1 M NaCl alone. When these MeJA solutions were combined with 0.2 M NaCl, respiration intensity was reduced by up to ~35%. Even stronger negative effects were observed for MEF and NaCl mixtures, with respiration rate dropping by up to ~57% at the highest concentrations of both treatments. Importantly, incubation in NaCl solutions alone caused significant upregulation in seed respiration intensity compared to the control only when 0.1 M salt was used (Figure 2A). Application of MeJA to seeds under NaCl stress exacerbated ion leakage evoked by NaCl, with the effect intensifying in proportion to the salt concentration (Figure 2B). A similar effect of pronounced seed damage compared to 0.1 M NaCl treatment was observed when seeds were simultaneously treated with salt and higher concentration of MeJA and inhibitor. Notably, combined treatment with 0.001 mM or 0.01 mM MeJA and 0.2 M NaCl resulted in a ~10% increase in seed damage compared to NaCl alone. Application of MeJA or MEF in the absence of NaCl did not cause seed damage (Figure 2B). In summary, although both MeJA and MEF treatments increased respiration without causing tissue damage, when applied during salt stress, they may potentiate the adverse effects induced by this stressor.

### 2.4. The Multifaced Influence of MeJA on Hemp Involves Modulation of Plant Morphology, Biometric Traits, and Photosynthesis-Related Parameters Under Salinity Stress

**Figure 3:**
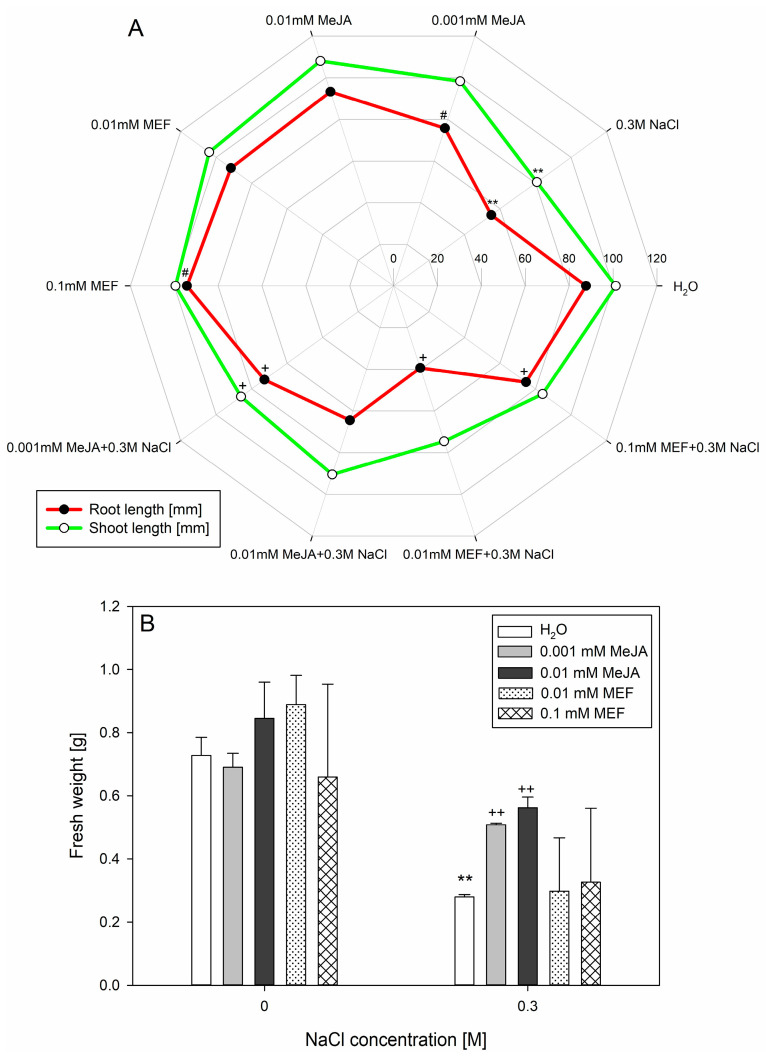
Effects of methyl jasmonate (MeJA) and mefenamic acid (MEF) on root and shoot length (**A**) and fresh weight (**B**) of 25-day-old hemp cultivated under salt stress conditions. Treatments included NaCl (0.3 M), MeJA (0.001 mM and 0.01 mM), MEF (0.01 mM and 0.1 mM), and their combinations. Values represent means ± SD. Statistical significance: NaCl vs. H_2_O ** *p* ≤ 0.01; MeJA/MEF vs. H_2_O ^#^ *p* ≤ 0.05; MeJA/MEF + NaCl vs. NaCl ^++^ *p* ≤ 0.01, ^+^ *p* ≤ 0.05.

A single treatment with 0.1 mM MEF promoted root growth of 25-day-old hemp, while 0.001 mM MeJA evoked the opposite effect. However, application of 0.001 mM MeJA to salt-stressed plants resulted in a significant increase in root length, by approximately 30 mm, when compared to seedlings treated with NaCl alone. A similar outcome was observed in plants subjected to NaCl and 0.1 mM MEF. In contrast, simultaneous application of salt and 0.01 mM JAs biosynthesis inhibitor suppressed root growth (Figure 3A).

In the upper part of the hemp plants, 0.001 mM MeJA reversed the inhibitory effect of salt on shoot elongation, which was not observed in MEF-treated plants or those treated with JAs or the inhibitor alone. No statistically significant differences in shoot length were also found among plants receiving salt plus 0.01 mM MeJA (Figure 3A). Exogenous MeJA at both tested concentrations nearly doubled the fresh weight of NaCl-stressed hemp, whereas MEF had no significant effect on biomass accumulation (Figure 3B).

**Figure 4:**
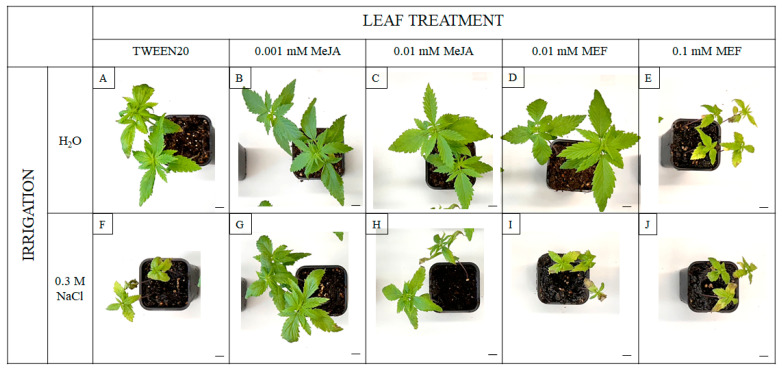
Representative morphology of 25-day-old hemp plants subjected to salt stress and treatments with MeJA and MEF. Plants were grown under control conditions (**A**), with 0.3 M NaCl alone (**F**), with exogenous 0.001 mM or 0.01 mM MeJA (**B**,**C**), 0.01 mM or 0.1 mM MEF (**D**,**E**), or with combinations of 0.3 M NaCl with MeJA (**G**,**H**) or MEF (**I**,**J**). Scale bar = 1 cm.

Visible symptoms of salinity stress and plant regulator treatments include not only growth modulation. Under salt stress, plants exhibited pronounced signs of abiotic stress, including leaf chlorosis and necrosis (Figure 4F). Comparable stress symptoms were observed in plants treated with 0.1 mM MEF, both alone and in combination with NaCl (Figure 4E,J). Notably, the deleterious visual effects of salinity were alleviated by application of 0.001 mM MeJA, resulting in improved plant appearance (Figure 4G). Collectively, these findings demonstrate that while salt stress suppresses growth and vitality of hemp, exogenous MeJA—particularly at lower concentrations—can partially mitigate the negative effects of the stressor, improving both root growth and overall plant condition. In contrast, MEF treatment exacerbated salinity-induced stress.

**Figure 5:**
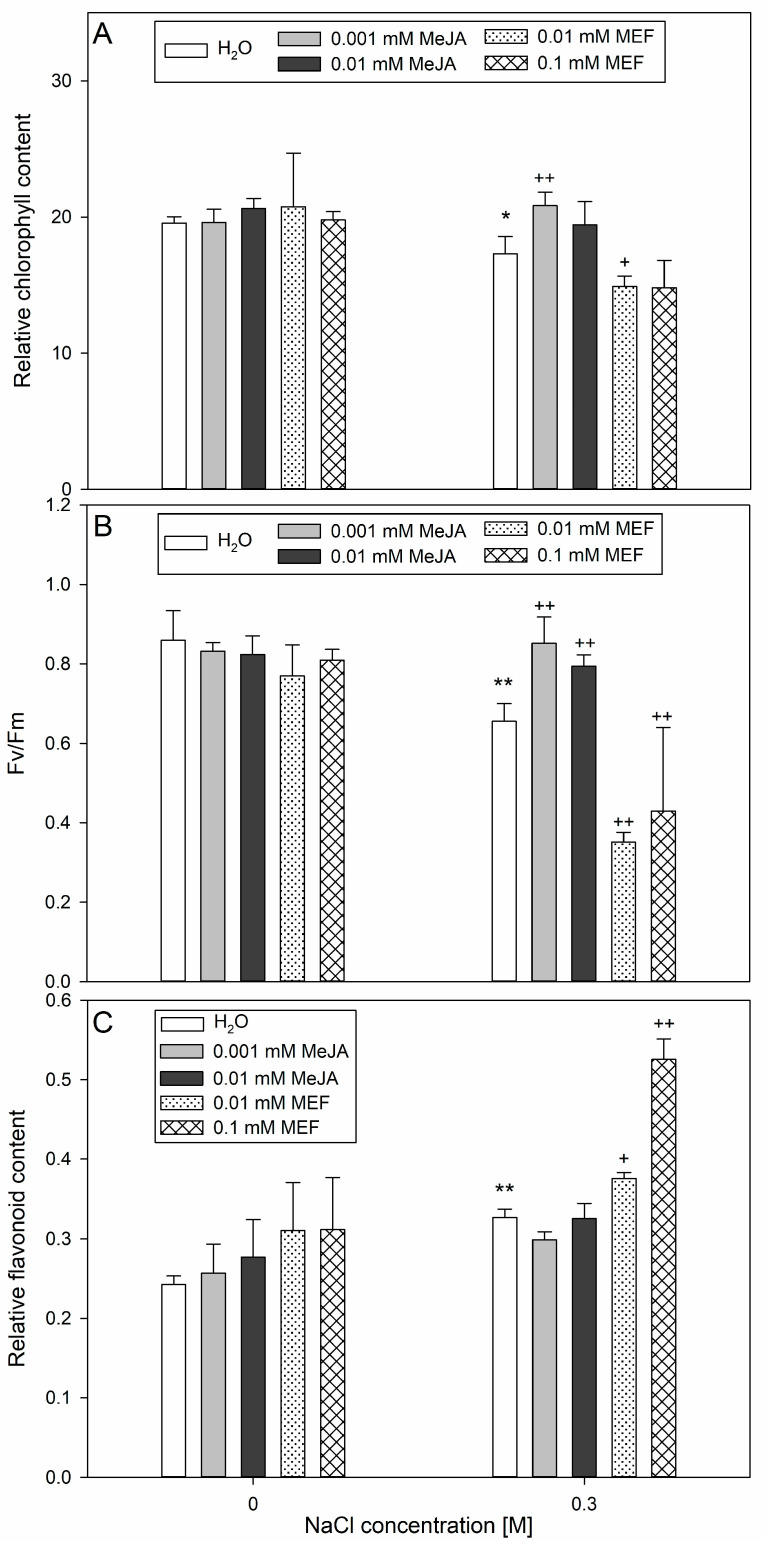
Effects of methyl jasmonate (MeJA) and mefenamic acid (MEF) on total chlorophyll content (**A**), F_v_/F_m_ (**B**), and flavonoid level (**C**) in leaves of 25-day-old hemp subjected to salt stress. Treatments included NaCl (0.3 M), MeJA (0.001 mM and 0.01 mM), MEF (0.01 mM and 0.1 mM), and their combinations. Values represent means ± SD. Statistical significance: NaCl vs. H_2_O ** *p* ≤ 0.01, * *p* ≤ 0.05; MeJA/MEF + NaCl vs. NaCl ^++^ *p* ≤ 0.01, ^+^ *p* ≤ 0.05.

Salt stress significantly affected the leaf chlorophyll content in hemp, reduced the efficiency of PSII and simultaneously increased the accumulation of flavonoids (Figure 5). Application of 0.001 mM MeJA led to an increase in chlorophyll content in the leaves of salinity-treated plants; in contrast, application of 0.01 mM MEF together with 0.3 M NaCl caused a significant decrease in leaf chlorophyll content (Figure 5A). Concerning photochemical efficiency, NaCl treatment moderately inhibited PSII activity as indicated by F_v_/F_m_ values ~0.68. However, application of 0.001 mM MeJA or 0.01 mM MeJA reversed this effect, stabilizing the F_v_/F_m_ parameter to nearly the control value (Figure 5B). Regardless of the concentration of MEF, when applied with salt, it further exacerbated this decline in photosynthetic efficiency. Combined NaCl and 0.01 mM MEF or 0.1 mM MEF treatment amplified the salt-induced increase in flavonoid content in leaves by approximately 16% and 40%, respectively, compared to salt stress alone (Figure 5C). However, none of the MeJA treatments, regardless of concentration, had a significant effect on flavonoid levels in seedlings, either in control or salt-stressed conditions (Figure 5C). Overall, these results indicate that while the salinity primarily impairs photosynthetic performance and triggers flavonoid accumulation, MeJA can alleviate the reduction in chlorophyll and PSII efficiency under stress. At the same time, MEF aggravates both pigment loss and salt-induced photosynthetic stress responses.

**Figure 6:**
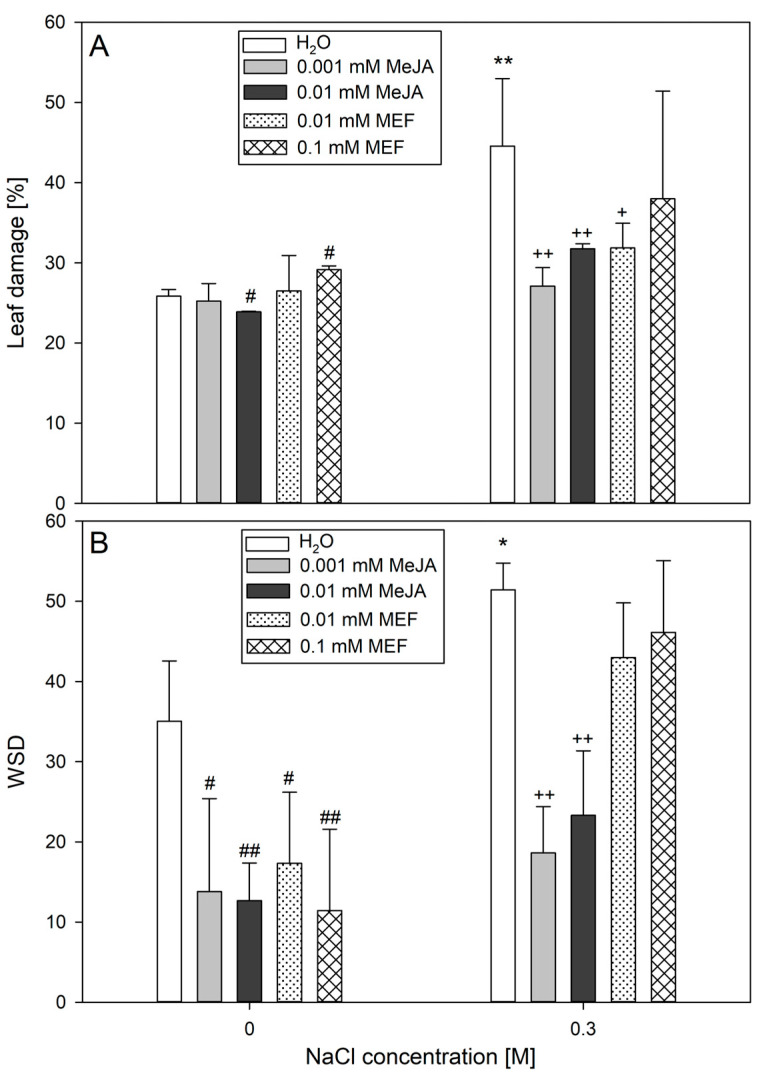
Effects of methyl jasmonate (MeJA) and mefenamic acid (MEF) on leaf damage (**A**) and WSD (**B**) in 25-day-old plants subjected to salt stress. Treatments included NaCl (0.3 M), MeJA (0.001 mM and 0.01 mM), MEF (0.01 mM and 0.1 mM), and their combinations. Values represent means ± SD. Statistical significance: NaCl vs. H_2_O ** *p* ≤ 0.01, * *p* ≤ 0.05; MeJA/MEF vs. H_2_O ^##^ *p* ≤ 0.01, ^#^ *p* ≤ 0.05; MeJA/MEF + NaCl vs. NaCl ^++^ *p* ≤ 0.01, ^+^ *p* ≤ 0.05.

At the subsequent stage of the study, the extent of leaf damage in plants subjected to MeJA or MEF treatment under salinity stress was assessed based on the electrolyte leakage assay (Figure 6A). In control plants, baseline membrane permeability resulted in ~25% electrolyte leakage, representing normal cellular turnover and minor handling damage. At higher concentrations, MeJA and MEF produced opposite effects, resulting in decreased and increased ion leakage, respectively. Application of 0.001 mM MeJA, 0.01 mM MeJA, or 0.01 mM MEF significantly reduced salt-induced leaf damage. The most notable protective effect was observed with the lowest MeJA concentration, which lowered NaCl-induced damage by approximately 40% compared to control value (Figure 6A). Analysis of leaf hydration parameters revealed that treatment with either MeJA or MEF solutions nearly halved the WSD index in leaves compared to the control, with the greatest reduction seen for 0.01 mM MeJA (Figure 6B). Under salinity conditions, MeJA at both tested concentrations greatly improved tissue hydration by reducing the WSD index almost threefold relative to salt-stressed plants. In contrast, no significant changes in tissue hydration were observed in seedlings co-treated with NaCl and MEF. Overall, these findings demonstrate that MeJA, particularly at lower concentrations, is effective in protecting hemp leaves from salt-induced leaf damage and water deficit, whereas MEF can limit leaf damage but exerts no effects on tissue hydration under stress.

## 3. Discussion

Despite extensive evidence in other species, JAs-mediated salt tolerance in hemp remains unexplored. In this study, we systematically evaluated the capacity of JAs to alleviate salinity stress in the Henola hemp cultivar during germination and early vegetative growth stages. Exposure to 0.1 M and 0.2 M NaCl significantly reduced germination rates (Supplementary Figure S1), consistent with osmotic stress impeding water uptake as the primary inhibitory mechanism [45]. These concentrations were thus selected for subsequent JAs-related experiments. Comparable findings by Hu et al. [46] reported similar germination and early developmental inhibition of industrial hemp under salt stress exceeding 0.1 M NaCl. Notably, complete germination inhibition at 0.3 M NaCl (Supplementary Figure S1) prevented its use in seed assays, although this concentration was necessary to elicit stress responses in 25-day-old seedlings (Supplementary Figure S2). This observation underscores a developmentally regulated shift in salt sensitivity critical for dissecting and comparing stress adaptation mechanisms across physiological stages. A similar developmental variation in salt tolerance was reported in *Medicago*, where germination-stage salt tolerance failed to predict resilience during vegetative growth [47].

Plants utilize a spectrum of salt-tolerance strategies including metabolic reprogramming, ion detoxification, and morphological plasticity, which can be further enhanced by exogenous priming agents, such as JAs, particularly MeJA. Effects of MeJA on germination are highly species-specific: it inhibits seed germination in tobacco [48], *Amaranthus caudatus* [49], and sunflower [50], but breaks dormancy in wheat [51], or promotes germination in *Cuscuta campestris* [52]. Here, both MeJA and MEF suppressed hemp germination under control conditions (Figure 1A). Strikingly, low-dose MeJA (0.001 mM) accelerated hemp germination under mild salinity (0.1 M NaCl) (Figure 1A), confirming a protective role during salt exposure. Conversely, application of the JAs biosynthesis inhibitor (0.1 mM MEF) worsened salt-induced germination delays, highlighting the crucial role of endogenous JAs homeostasis for optimal seed performance. The observed species-specific dosing and timing effects align with prior reports—for example *Ocimum bacilicum* L. responds to sub-micromolar MeJA doses (0.1 µM or 0.01 µM MeJA) to overcome inhibition induced by 0.1 M or 0.2 M NaCl [53], whereas okra requires micromolar concentrations (50–500 μM MeJA) [54]. The ability of JAs to restore germination under sublethal stress is paralleled in other crops, where they induce protective pathways including HEAT SHOCK PROTEINS and antioxidant enzymes that preserve mRNA integrity, protein folding, and membrane stability [55].

Seed respiration serves as a vital nexus integrating energy metabolism, reserve mobilization, and stress-acclimatization pathways to support successful germination and seedling establishment. Exogenous MeJA (0.25 µM) has been shown to enhance wheat leaf respiration under drought [56], underscoring its role in compensatory metabolism. In hemp, mild salinity (0.1 M NaCl) induced a similar increase in seed respiration (Figure 2A), reflecting the activation of defense-related metabolic pathways to meet enhanced energy demands and synthesize protective compounds. At higher salinity (0.2 M NaCl), this respiratory stimulation diminished and seed damage was evident (Figure 2B), indicating a threshold beyond which adaptive respiration is impaired. The progressive seed damage with rising NaCl concentration (Figure 2B) likely results from salt-induced lipid peroxidation and membrane destabilization, paralleling observations in durum wheat, where 0.15 M NaCl elicited 40% tissue injury through elevated ROS formation [57]. MeJA or MEF treatments further elevated seed respiration under non-saline conditions; however, this effect was negated by salt (Figure 2A), indicating complex JA-salinity interactions during early seed physiology. Together with improved germination (Figure 1) and absence of damage following co-treatment with 0.001 mM MeJA and 0.1 M NaCl (Figure 2B), these results suggest that reductions in respiration under combined stress are transient. This interpretation is strengthened by strong respiratory inhibition by MEF at all salinity levels and pronounced suppression under 0.2 M NaCl with MeJA (Figure 2B), likely reflecting severe structural seed damage caused by intense salt stress.

At the seedling stage, 0.1 M NaCl caused severe hemp growth inhibition (Figure 1), probably via impaired water uptake and ion toxicity [58]. This magnitude of root length reduction (~50%) was observed in canola under similar salinity [59], indicating comparable tolerance thresholds among oilseed plants. Although MeJA co-application with 0.1 M NaCl did not fully restore total biomass, MEF exacerbated salt stress symptoms (Figure 1B,C), emphasizing the indispensable function of endogenous JAs in maintaining growth under salt stress [60]. The effects of JAs treatments vary widely by species and context; for example, MeJA intensifies the negative effect of NaCl on root elongation in alfalfa but partially rescues biomass losses in shoots and roots [61], while in maize it promotes growth under drought when applied as seed priming [62]. We demonstrate stage-dependent sensitivity in hemp: a single application of 0.001 mM MeJA under 0.1 M NaCl fully restored root and shoot lengths (Figure 3A), increased fresh weight of 25-day-old seedlings (Figure 3B), and improved plant morphology (Figure 4). The growth-promoting effect of MeJA is likely linked to activation of defense pathways enhancing water and mineral uptake [63], optimizing water potential and water use efficiency, comparable to effects described in rapeseed under salinity [44]. Intriguingly, under non-stressed conditions, 0.001 mM MeJA reduced root length, consistent with JAs acting as “danger” signals suppressing growth—a response also elicited by 0.1 mM MEF. Parallel findings in *Anchusa italic* showed enhanced root growth and biomass under combined 120 μM MeJA and 0.1 M NaCl through increased proline, chlorophyll content, and lowered cellular water potential [64]. In hemp, MeJA treatment increased chlorophyll content (Figure 5A) and restored PSII efficiency (F_v_/F_m_) under salt stress, effects abolished by MEF (Figure 5B), illustrating JAs’ protective role for photosynthetic machinery. These observations align with reports of MeJA accelerating photosynthetic recovery under heat stress in wheat by safeguarding PSII and stabilizing the D1 protein [65], which is essential for PSII repair [66]. MeJA also upregulates antioxidant enzymes (superoxide dismutase, catalase, peroxidase) that shield photosynthesis under severe salinity stress in tomato [67]. It has been documented that MeJA reduces oxidative damage by modulating antioxidant-related gene expression, decreasing ROS burden in *Brassica napus* [68]. Similarly, in rice, severe salt stress-induced oxidative damage due to ROS accumulation was mitigated in OPDA-deficient mutants via enhanced JAs biosynthetic pathway-linked ROS scavenging [69]. Furthermore, MeJA prevents salt-induced chlorophyll loss in sorghum [33], likely by stimulating chlorophyll biosynthesis via 5-aminolevulinic acid [70]. In rice, salt-driven reductions in F_v_/F_m_ correlate with chlorophyll degradation, impaired photosynthesis, and chloroplast membrane damage [71,72]. Under severe salinity (0.3 M NaCl), hemp demonstrated marked leaf injury, elevated WSD (Figure 6), and senescence symptoms (Figure 4), along with reduced chlorophyll (Figure 5A), PSII inefficiency (Figure 5B), and increased flavonoids (Figure 5C), reflecting adaptive chlorophyll loss to reduce photoinhibition risk coupled with flavonoid antioxidant protection [73,74]. JAs may directly stimulate flavonoid biosynthesis under salt stress, as documented in *Dendrobium* leaves [75]. The ability of MeJA to reverse NaCl-induced declines in chlorophyll, PSII efficiency, leaf damage, and WSD highlights its efficacy in mitigating salinity effects in hemp by stabilizing tissue hydration and enhancing photosynthesis. MEF’s antagonistic effects further confirm the protective role of endogenous JAs under salt stress. Consistent JAs-mediated salt-stress alleviation has been reported in grapevine, where MeJA increases photosynthetic pigments, flavonoids, proline, and membrane stability [76], as well as in wheat, where JA limits Na^+^ accumulation and improves photosynthesis and seedling morphology [77].

**Figure 7:**
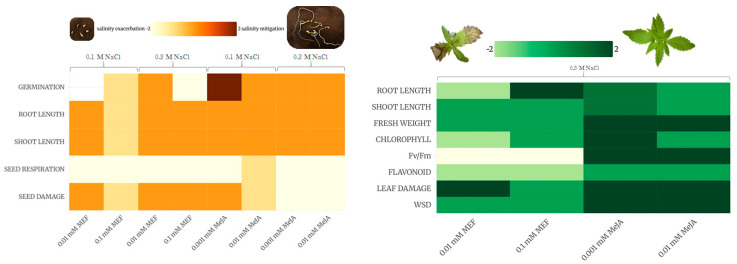
Heat maps representation of MeJA and MEF efficacy in mitigating the effects of salinity stress on hemp during seed germination (**left panel**) and early growth (**right panel**). A color gradient ranging from light yellow (or green) to brown (or dark green) indicates increasing mitigation effect of salinity stress (−2—salinity exacerbation; 2—salinity mitigation). For comprehensive analysis and interpretation of treatment effects, refer to the Discussion section.

The evaluated traits showed considerable variation across developmental stages, indicating that hemp’s salt sensitivity is age-dependent and underscoring the importance of multi-stage assessment. Heatmap analyses (Figure 7) reveal profound impacts of JAs modulation or inhibition on hemp performance under various salinity regimes. Germination was highly susceptible to 0.1 mM MEF at both NaCl levels, leading to further reductions in germination and seedling growth relative to salt alone, whereas low dose MeJA (0.001 mM) effectively restored germination vigor. Seed respiration, impacted by all treatments, was protected from salt damage by 0.001 mM MeJA. During vegetative development under mild salinity, MeJA enhanced nearly all measured traits, demonstrating potent mitigation of salt stress, while MEF exacerbated almost all these adverse effects at both concentrations. These findings demonstrate, for the first time, the critical role of JAs in mediating salt stress tolerance in hemp, highlighting MeJA application as a promising, sustainable, and eco-friendly approach to enhance hemp productivity under abiotic stress. Concentration-specific and timing-dependent effects enable developmental stage-targeted management protocols for optimized resource use and economic returns. This work provides a foundation for expanding hemp cultivation aimed at high-value products by mitigating yield losses though hormonal modulation.

## 4. Material and Methods

### 4.1. Plant Material and Experimental Design

The material used in the study was *Cannabis sativa* Henola variety, with seeds obtained from the Institute of Natural Fibers and Medicinal Plants in Poznań. The Henola cultivar is distinguished by a shorter vegetative period and a seed yield that is approximately twice that of other hemp varieties [78]. This study employed a two-phase factorial experimental design to investigate JAs’ modulation of salt stress responses in hemp during germination (Petri dish assay, Supplementary Figure S3) and early seedling development (pot cultivation, Supplementary Figure S4A). The first phase established optimal NaCl concentrations for inducing measurable stress responses. The second phase examined how MeJA and MEF influence plant responses under saline conditions. MEF was included to modulate the physiological response induced by JAs [79]; as we demonstrated previously, plants treated with 0.1 mM MEF showed reduced MeJA content without exhibiting phytotoxicity [80]. Each treatment was tested under various salt stress conditions to evaluate concentration-dependent and stress-specific responses. The design incorporated multiple control strategies: negative controls (no stress, water treatment), positive controls (salt stress only), and treatment-specific controls (individual MeJA/MEF applications).

### 4.2. Germination Test

To identify the salinity threshold that induces a physiological response in hemp seeds, an initial germination assay was conducted. Seeds were rinsed three times with distilled water before being handled aseptically in a laminar flow hood. For each treatment, 100 seeds in 5 replicates were placed on filter paper within Petri dishes containing 10 mL of NaCl solutions at concentrations of 0.05 M, 0.1 M, 0.2 M, and 0.3 M. In the second stage, the effects of JAs on hemp seed germination under saline conditions were examined. Germination assays were performed using the most effective NaCl concentrations identified in the preliminary tests (0.1 M and 0.2 M), supplemented with either MeJA (0.001 mM and 0.01 mM) or MEF (0.01 mM and 0.1 mM). For all treatments, seeds incubated in distilled water served as controls. All Petri dishes were placed in a climate chamber (MLR-352H-PE, Panasonic, Osaka, Japan) under controlled environmental conditions (darkness, 22 °C, and 50% relative humidity). After 7 days, germinated seeds were counted. Additionally, for experiments involving JAs, root and shoot lengths of seedlings were measured.

### 4.3. Seed Respiration Rate

The rate of seed respiration was measured using a Q-S151 gas analyzer (Qubit Systems Inc., Kingston, ON, Canada) by continuously monitoring CO_2_ evolution based on nondispersive infrared absorption; the resulting decrease in infrared transmission is proportional to CO_2_ concentration. For each experimental condition, ~0.5 g of seeds—previously incubated for 24 h in the respective test solutions (NaCl, MeJA, MEF, and their combinations, the same as for germination test)—were placed into a 10 mL darkened measurement chamber. Seeds incubated in distilled water served as controls. CO_2_ production was continuously recorded over 5 min period at room temperature. Respiration rates (µmol CO_2_ kg^−1^ s^−1^) were calculated from the slope of CO_2_ accumulation over time. All data are reported as mean respiration rates derived from six biological replicates.

### 4.4. Hemp Cultivation and Plant Treatments

To assess the effect of salinity on vegetative growth, seeds were first soaked in distilled water for 24 h before sowing. They were subsequently sown in 0.2 L pots containing standardized soil substrate (Canna Terra Professional, pH 5.5–6.5; EC 1.1–1.3 mS/cm; the content of mineral fertilizers NPK (12/14/24): 1.5 kg/m^3^), representing a commercial growing medium with controlled baseline nutrient availability and consistent physical properties. Plants were grown for 10 days under controlled phytotron conditions (16 h light at 40 µmol m^−2^ s^−1^ PAR, 8 h darkness, 22 °C, 50% relative humidity). On the 10th day, plants were separated into 5 groups, each comprising 10 individuals, and irrigated with 10 mL of either distilled water (control) or NaCl solutions at concentrations 0.05 M, 0.1 M, 0.2 M, and 0.3 M. Irrigation treatments were repeated on days 13 and 15. For the following 10 days, plants in all treatments received equal volumes of water, adjusted according to developmental requirements (Supplementary Figure S4A).

In a subsequent experiment examining the impact of JAs under saline conditions, plants were grown for 10 days as previously described and then assigned to 10 groups of 10 plants. These groups were subjected to three NaCl treatments on days 10, 13, and 15. Concurrently, MeJA (0.001 mM and 0.01 mM) or MEF (0.01 mM and 0.1 mM), dissolved in 0.05% Tween 20, were applied to leaves and shoot apices using sterile brushes (Supplementary Figure S4B). Control plants received 0.05% Tween 20 only. Following these treatments, plants were maintained for an additional 10 days and subsequently analyzed with a fluorimeter and Dualex. At harvest, plants were carefully removed from pots, and roots were washed. Shoot and root lengths were measured, and fresh biomass was determined. The oldest leaves—the first true leaves that developed after cotyledons and were treated with growth regulators—were collected to assess tissue hydration and injury.

### 4.5. Photosynthetic-Related Parameters

Physiological measurements were conducted on the oldest leaves of 25-day-old hemp using a portable fluorometer and a Dualex sensor. Chlorophyll a fluorescence was assessed with a Handy-PEA chlorophyll fluorometer (Hansatech Instruments, King’s Lynn, Norfolk, UK) following 30 min of dark adaptation. Fluorescence was induced by a 1-s saturating light pulse at 3000 µmol m^−2^ s^−1^, and the instrument automatically computed the maximum quantum yield of photosystem II (F_v_/F_m_). Leaf chlorophyll and flavonoid concentrations were determined using a Dualex Scientific sensor (Dualex Scientific, Force-A Co., Orsay, France), providing non-destructive, in situ measurements of these key parameters.

### 4.6. Water Saturation Deficit

The two oldest leaves from each plant were harvested and immediately weighed to determine water saturation deficit (WSD) following the method outlined by Turner [81]. To achieve full turgor, leaf samples were subsequently rehydrated by floating them on distilled water in darkness for 24 h. After blotting dry, the leaves were weighed again. Each sample was then oven-dried at 105 °C for 24 h, and its dry mass was recorded. WSD was calculated for each sample and expressed as a percentage. All data are presented as mean values from six replicates.

### 4.7. Tissue Damage

Damage to both seeds and leaves was evaluated by measuring exudate leakage via a conductometric assay—a method based on the measurements of membrane integrity loss through ion efflux from damaged cells. Seeds were incubated for 24 h in the designated test solutions. For vegetative-stage plants, five 5-mm leaf discs were excised from the oldest leaves. All samples were rinsed with deionized water to remove surface ions, then transferred into 20 mL of deionized water and incubated at 21 °C for 4 h. The initial electrical conductivity (EC_1_) of the solution was recorded using a conductometer. Samples were then boiled for 15 min in a water bath to induce complete membrane rupture, cooled to room temperature, and the conductivity was measured again (EC_2_). Membrane destabilization (%) was calculated as (EC_1_/EC_2_) × 100%, where EC_2_ represents total ion release. Results are presented as the mean of six replicates.

### 4.8. Statistical Analyses

Statistical analyses and data visualization were performed using Microsoft Excel and SigmaPlot. All results are reported as mean ± standard deviation (SD). Group differences were assessed by one-way analysis of variance (ANOVA), followed by Tukey’s post hoc test for pairwise comparisons. Prior to performing ANOVA and post hoc test, the data sets were evaluated for normality (Shapiro–Wilk test) and homogeneity of variances (Levene’s test). Statistical significance was defined at *p* ≤ 0.05 and *p* ≤ 0.01 and indicated on figures with symbols described in each legend.

## Data Availability

The original contributions presented in this study are included in the article/Supplementary Materials. Further inquiries can be directed to the corresponding author.

## Supplementary Materials

The following supporting information can be downloaded at: https://www.mdpi.com/article/10.3390/plants14182864/s1, Supplementary Figure S1. Impact of salinity on germination-related processes in hemp. Supplementary Figure S2. Growth-related parameters in 25-day-old hemp seedlings grown under salinity stress. Supplementary Figure S3. Diagram illustrating the experimental setup for assessing the effects of MeJA and MEF on hemp seed germination under saline conditions. Supplementary Figure S4. Experimental design diagram showing treatment variants (A) and timing of solution applications (three-times) (B) for evaluating the effects of MeJA and MEF on early seedling development under saline conditions.
