# Supplementary material for: Jasmonate-Mediated Mitigation of Salinity Stress During Germination and Early Vegetative Development in Hemp
Source: Plants (Basel). 2025 Sep 15;14(18):2864. doi: 10.3390/plants14182864 (PMC12473429; doi:10.3390/plants14182864)
Supplement: Supplementary file 1 [file plants-14-02864-s001.zip › plants-3855191-supplementary.pdf]

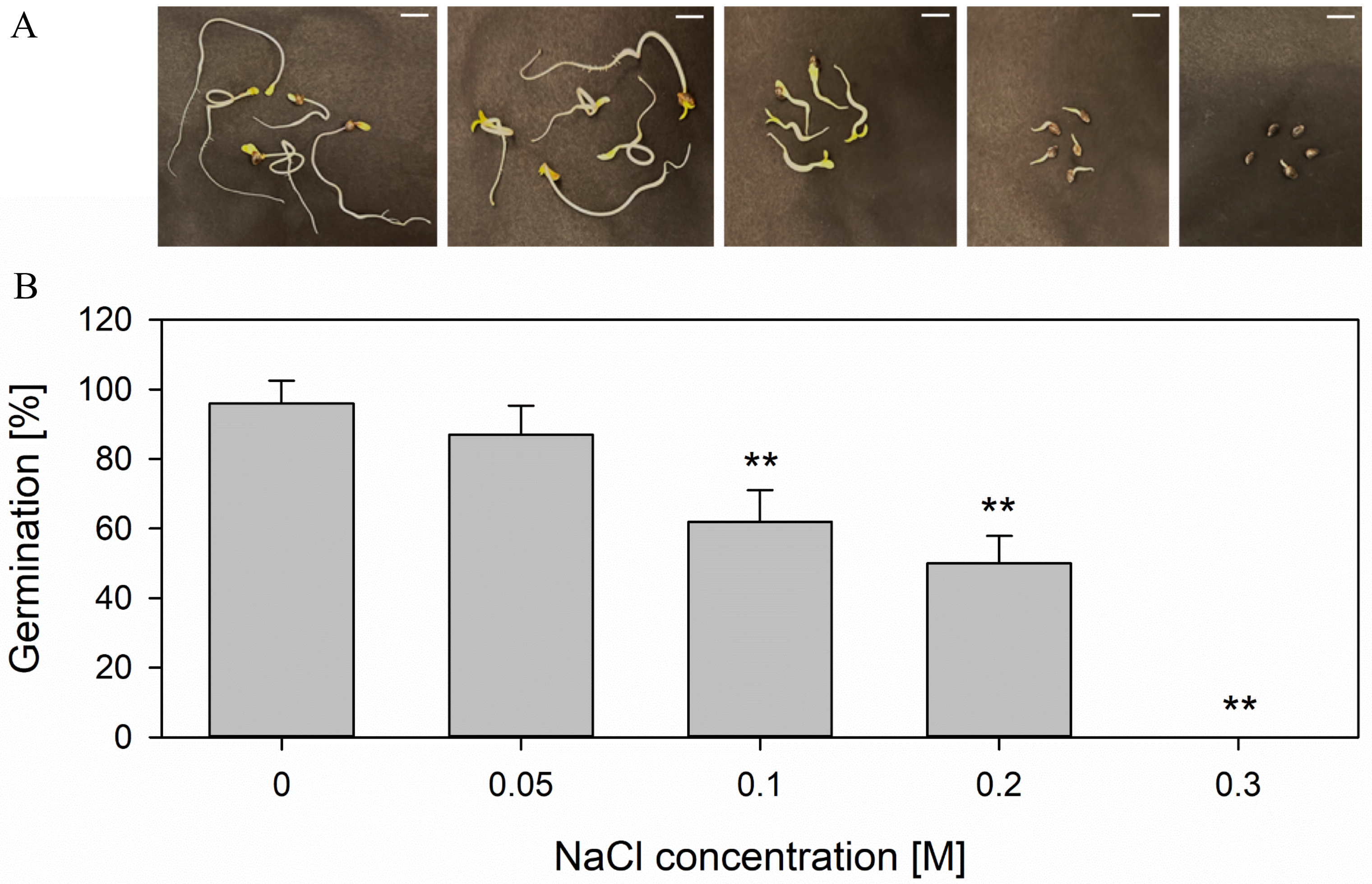


**Supplementary Figure S1. Impact of salinity on germination-related processes in hemp.**

Seeds were incubated in solutions with increasing NaCl concentrations (0.05 M, 0.1 M, 0.2 M, and 0.3 M) and water (control). Photographs show seedlings and seeds on day 7 of the germination test. Scale bar = 1 cm (A). The percentage of germinated seeds was recorded on day 7. Values are presented as means ± SD (B). Statistically significant differences between NaCl treatments and control are indicated ($^{**}p \leq 0.01$).

| | NaCl [M] | | | | |
|---|---|---|---|---|---|
| | $H_2O$ | 0.05 | 0.1 | 0.2 | 0.3 |
| Root length [mm] | 13.97 ± 3.11 | 8.86 ± 2.99$^{**}$ | 9.64 ± 2.38$^{**}$ | 7.68 ± 2.64$^{**}$ | 5.62 ± 2.26$^{**}$ |
| Shoot length [mm] | 11.64 ± 2.05 | 10.44 ± 1.70 | 10.22 ± 2.15 | 9.03 ± 1.90$^{**}$ | 7.64 ± 1.27$^{**}$ |

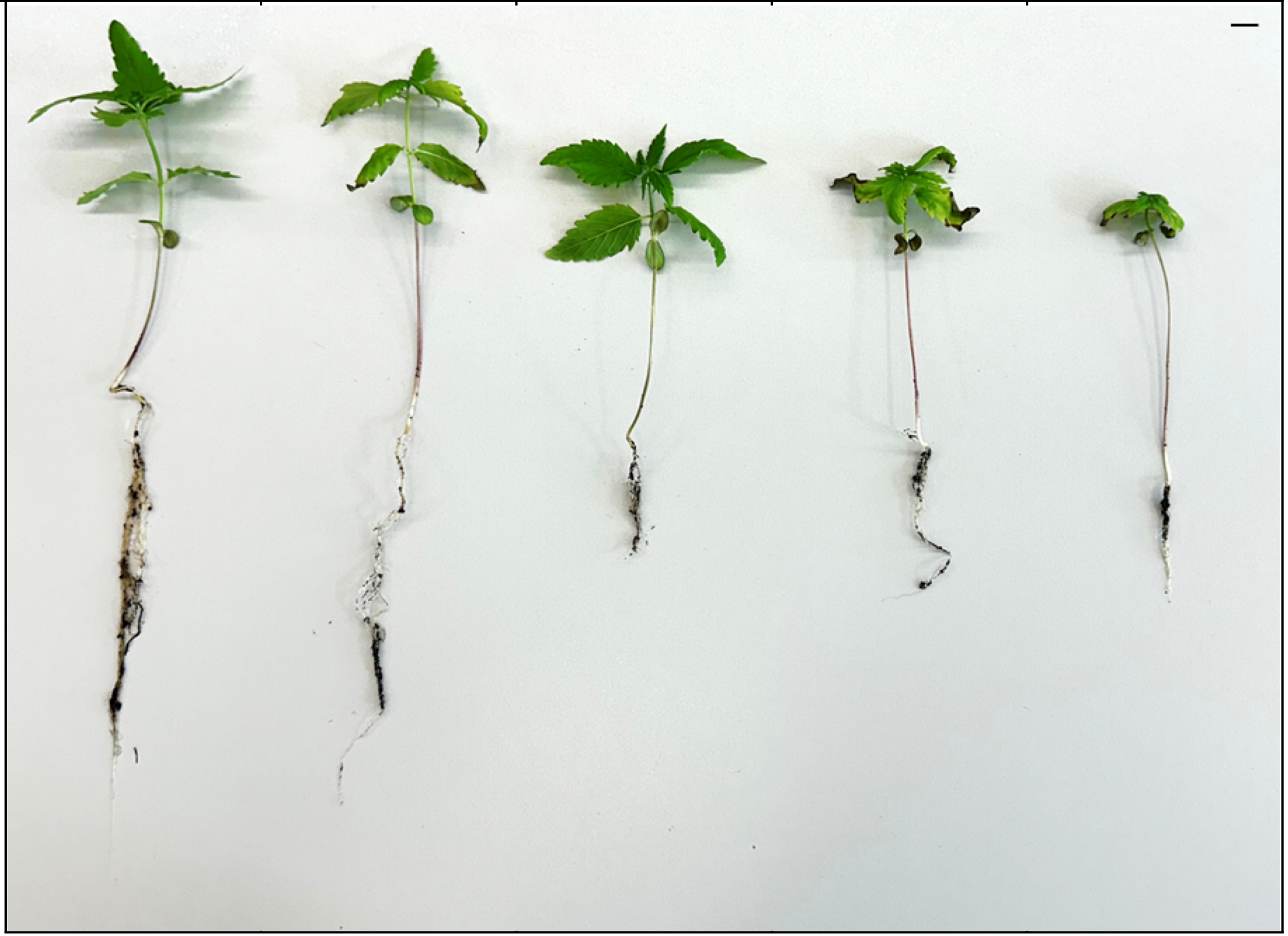

**Supplementary Figure S2. Growth-related parameters in 25-day-old hemp seedlings grown under salinity stress.** Root and shoot length are presented as means ± SD. Statistically significant differences between NaCl treatments and control are indicated ($^{**}p \leq 0.01$). Photographs show seedlings cultivated in the presence of increasing NaCl concentrations (0.05 M, 0.1 M, 0.2 M, and 0.3 M) and water (control). Scale bar = 1 cm.

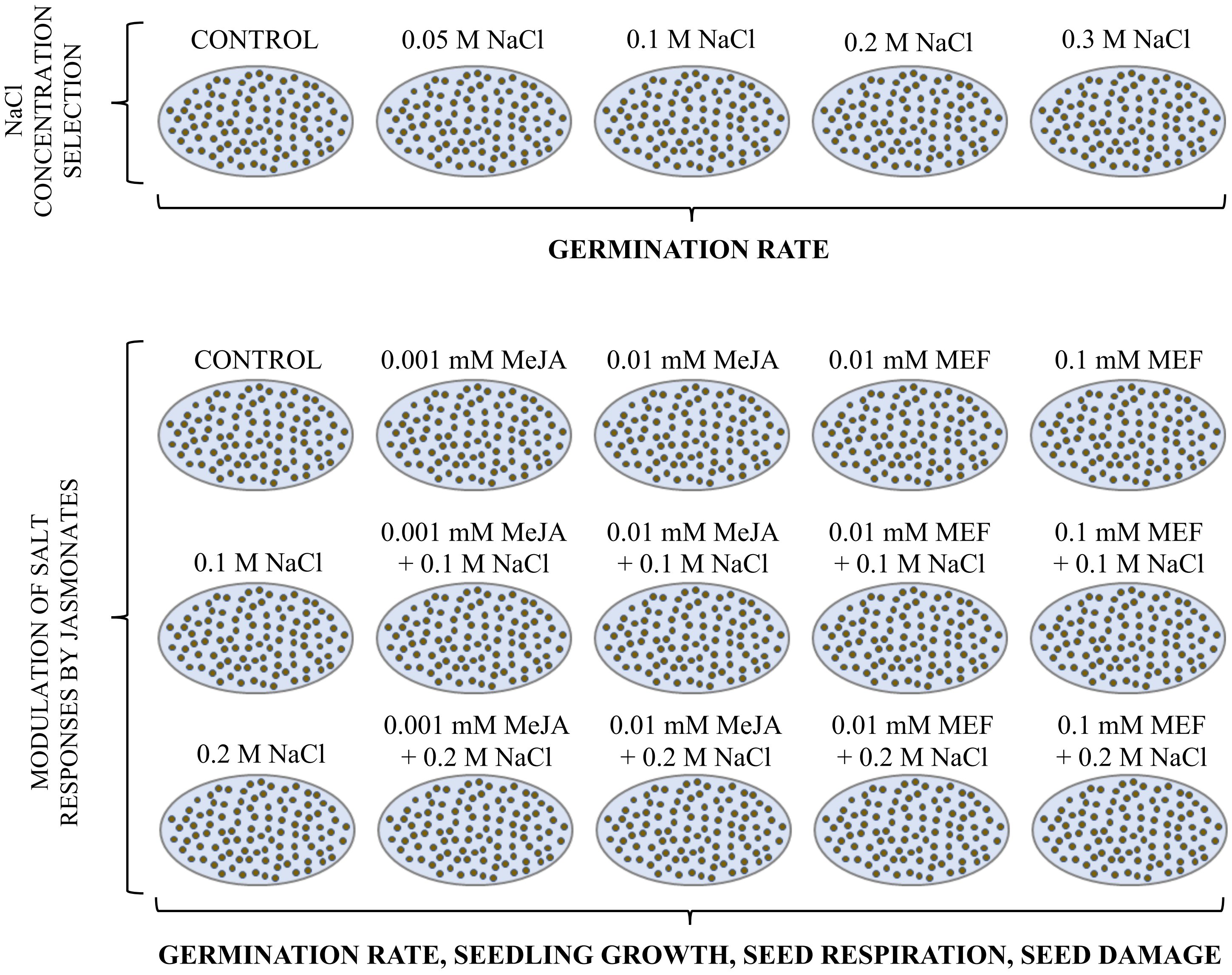


**Supplementary Figure S3. Diagram illustrating the experimental setup for assessing the effects of MeJA and MEF on hemp seed germination under saline conditions.** Hemp seeds were evenly spaced on Petri dishes containing filter paper soaked in treatment solutions. Seeds incubated in water served as the control. Each dish is labeled above with its solution composition and concentration. For details, see the Material and methods section.

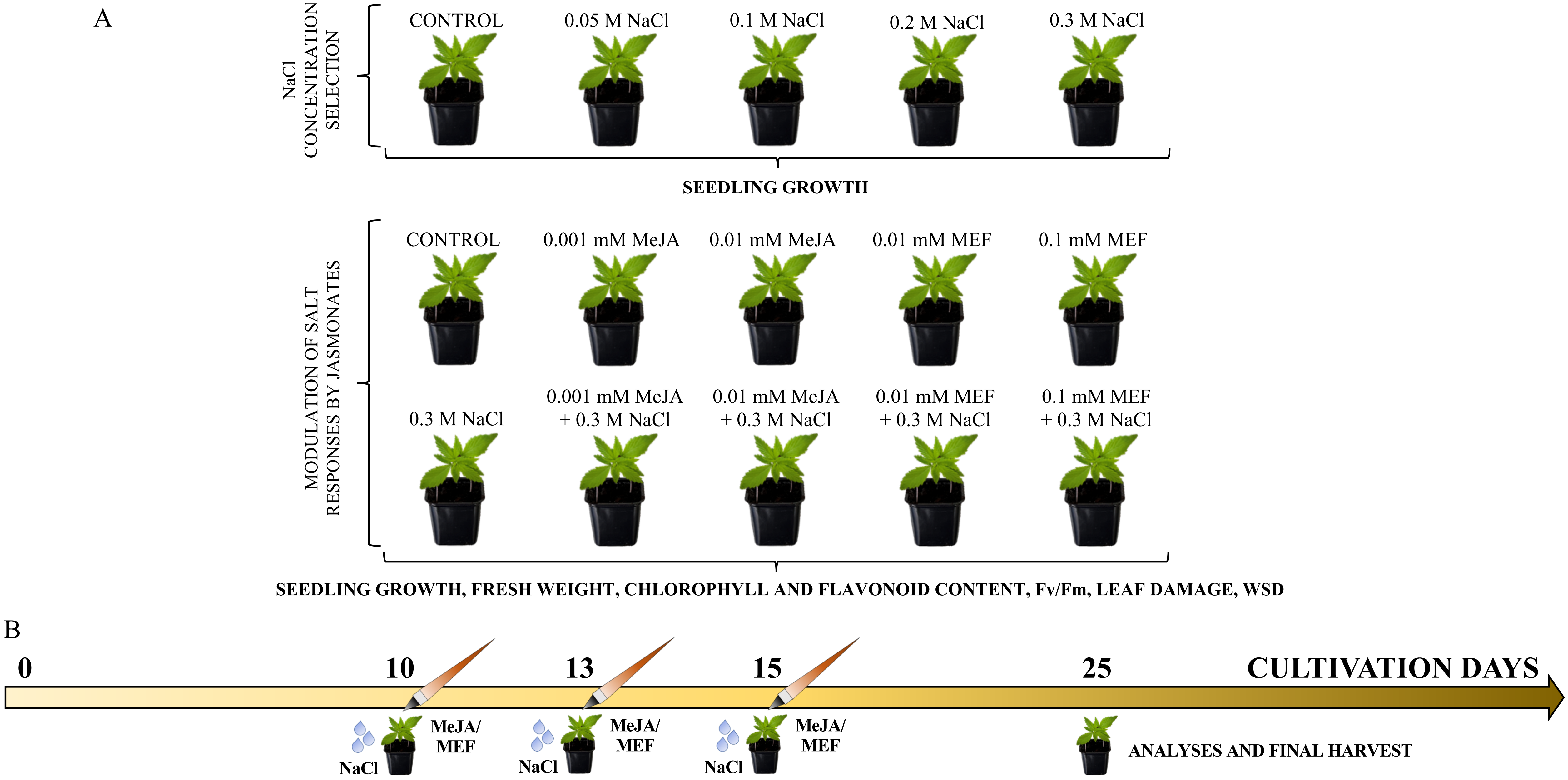


**Supplementary Figure S4. Experimental design diagram showing treatment variants (A) and timing of solution applications (three-times) (B) for evaluating the effects of MeJA and MEF on early seedling development under saline conditions.** For details, see the Material and methods section.
